# A Rare Twist of the Forgotten Disease: A Case of *Fusobacterium necrophorum* Sepsis with Portomesenteric Thrombosis and a Review of the Literature

**DOI:** 10.1155/2021/6699867

**Published:** 2021-05-27

**Authors:** Nicholas Lazar, Kamil Sardarli, Zaid Imam, Majd Khasawneh, Ismail Hader

**Affiliations:** ^1^Department of Internal Medicine, Beaumont Health, Royal Oak, MI, USA; ^2^Division of Gastroenterology and Hepatology, Department of Internal Medicine, Beaumont Health, Royal Oak, MI, USA; ^3^Division of Pulmonary Medicine and Critical Care, Department of Internal Medicine, University of Florida, Gainesville, FL, USA; ^4^Division of Gastroenterology and Hepatology, Sunrise Health Graduate Medical Education (GME), Las Vegas, NV, USA

## Abstract

Abdominal variants of Lemierre's syndrome presenting with pylephlebitis are rare, and the role of anticoagulation in treatment is controversial. We hereby report a case of pylephlebitis secondary to *F. necrophorum* bacteremia in a 57-year-old female originating from bacterial translocation secondary to colitis, who developed a favorable outcome with prompt treatment with antibiotics and anticoagulation. We also perform a literature review on similar cases in the literature and discuss management options of this rare but potentially fatal complication.

## 1. Introduction

Lemierre's syndrome, known as the “forgotten disease” is defined by septic thrombophlebitis of the internal jugular vein in the setting of *Fusobacterium necrophorum* infection. Pylephlebitis or septic thrombophlebitis of the portal venous system is a rare complication of appendicitis, cholecystitis, pancreatitis, diverticulitis, and other intra-abdominal infections [1]. It presents with fever, abdominal pain, nausea, and vomiting and carries a mortality ranging between 25–80%, and early recognition is key to improved outcomes [1–4]. Abdominal variants of Lemierre's syndrome with pylephlebitis are exceedingly rare and present management controversies, particularly regarding anticoagulation [4,5]. Hereby, we report a case of pylephlebitis secondary to *F. necrophorum* bacteremia and review relevant literature on this complication.

## 2. Case Report

A 57-year-old female with prior history of nonischemic cardiomyopathy and alcohol abuse was admitted to our facility with a three-day history of myalgias and chills. Accompanied by her husband, he corroborated that she became increasingly confused over the same duration. She had no prior history of cirrhosis or chronic pancreatitis. A review of systems was otherwise negative. She had no recent dental work or procedures. On exam, she was ill-appearing with no abdominal tenderness or guarding, a normal cardiopulmonary examination, and negative meningeal signs. She was febrile to 39.1°C (102.4°F), tachycardiac to 113 beats/minute, had a blood pressure of 113/80 mmHg, and was saturating well on room air.

Initial labs revealed a neutrophil count of 9,100 cells/mm [3], platelet count of 91,000 cells/mm [3], and lactic acid of 1.0 (normal: <2) mmol/L. Mild elevations in her aspartate aminotransferase (AST) and alanine aminotransferase to 55 (normal: 10–37) U/L and 62 (normal: 8–37) U/L, respectively, were noted with a normal alkaline phosphatase level of 58 (normal: 30–110) U/L. A chest X-ray and urinalysis were normal, and blood cultures were obtained. The patient rapidly deteriorated, became hypotensive and was resuscitated with isotonic fluid boluses, started on broad spectrum antibiotics with intravenous vancomycin and meropenem given a prior history of allergy to penicillin, and admitted to the intensive-care unit.

**Figure 1:**
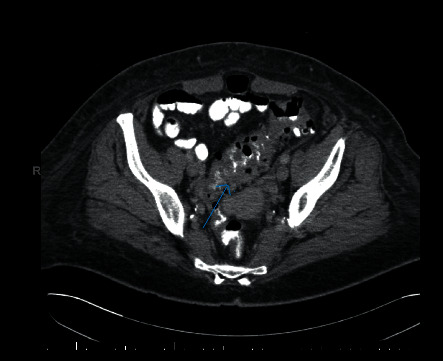
Axial computed tomography of the abdomen and pelvis demonstrating sigmoid colonic thickening and mild sigmoid colitis with no evidence of perforation on day 2 of hospitalization (blue arrow).

On her second day of admission, her mentation improved but she developed profuse, watery diarrhea and lower abdominal pain. *Clostridium difficile* testing and stool cultures were obtained and were negative. A computed tomography of her abdomen and pelvis with intravenous and oral contrast showed mild sigmoid colitis but no definite evidence of diverticulitis and cholelithiasis with no pericholecystic fluid (Figure 1). On day 2 of admission, the blood cultures returned positive for a Gram-negative bacillus identified on day 4 as *F. necrophorum*. A progressive elevation of her liver chemistries to ALP of 415 U/L, AST of 154 U/L, ALT of 134 U/L, and total bilirubin of 1.5 mg/dl prompted evaluation with a magnetic resonance pancreaticography (MRCP) for a biliary source of infection. Her common bile duct was normal in diameter at 7 mm with no filling defects, no extra- or intrahepatic biliary duct dilatation, and no pericholecystic inflammation or evidence of pancreatitis noted. However, the MRCP identified nonocclusive thrombosis of the proximal superior mesenteric vein and a few right portal vein branches with adjacent impaired hepatic perfusion. Given these findings and concern for focal hepatic tissue infarction, anticoagulation with intravenous heparin was initiated for a total of 72 hours titrated to a goal activated partial thromboplastin time (aPTT) of 60–80 seconds before transitioning to apixaban 5 mg twice daily.

With continued supportive care and antibiotic therapy, the patient improved gradually, her symptoms resolved, and she was discharged from our facility to complete a 14-day course of ertapenem. Additionally, she was treated with apixaban 5 mg twice daily for 3 months for her portomesenteric thrombosis. Her treatment course was uneventful, and no bleeding events occurred.

To date, the reported patient had two subsequent episodes of sigmoid diverticulitis 1 year later, the latter of which was complicated by perforation requiring a lower anterior resection, ileostomy placement, and subsequent reversal. She recovered well from her operations and has had no recurrent portal, mesenteric, or venous thromboses.

## 3. Discussion

*Fusobacterium necrophorum* is a commensal Gram-negative bacillus colonizing the respiratory, gastrointestinal, and female genital tract [6] and implicated as the causative agent of Lemierre's syndrome manifesting with septic internal jugular vein thrombophlebitis and often with septic pulmonary emboli [6, 7]. Pylephlebitis, or suppurative thrombophlebitis of the portomesenteric venous system, is a rare complication of intra-abdominal infections and is hardly ever a sequela of *Fusobacterium spp*. septicemia [4]. We hereby report a case of septic portomesenteric thrombosis secondary to *Fusobacterium necrophorum* bacteremia in the setting of sigmoid colitis to add to a total of 21 cases of *Fusobacterium*-*spp.*-infection-related pylephlebitis reported in the literature [8–27].

Clinically, pylephlebitis presents with fever, abdominal pain, hepatosplenomegaly, and, less commonly, ascites [4]. Neutrophilic leukocytosis and liver chemistry disturbances are commonly encountered in pylephlebitis from all bacterial etiologies [3, 4]. In reported cases of *Fusobacterium-*associated pylephlebitis (Table 1); liver enzyme abnormalities occurred in 16 (72.7%) patients, including the reported case. The median patient age in *Fusobacterium-spp.*-associated pylephlebitis was 52 years (interquartile range (IQR): 36–63) similar to a case series of patients with pylephlebitis from other bacterial etiologies and the age of the reported patient [5].

In *Fusobacterium-spp.*-associated pylephlebitis, hepatic abscesses (27%) and oropharyngeal infections (13.6%) represented the most common identified primary infection site, and no clear primary infection site was identified in 36.4% of patients (Table 1). This contrasts with pylephlebitis from other bacterial etiologies whereby diverticulitis, pancreaticobiliary etiologies, and intra-abdominal abscesses represent the most common identified primary infection sites [4, 5]. In the reported case, bacteremia from sigmoid colitis represents the likely source of infection in the absence of an alternative site and the patient's diarrhea.

Recent or remote abdominal surgery and immunosuppression have been reported as associated conditions with the development of pylephlebitis; however, pylephlebitis can occur in previously healthy individuals as well, highlighting the importance of a high index of suspicion in patients with intra-abdominal pathologies or *Fusobacterium spp.* bacteremia [3, 9, 12, 13, 15, 16, 22]. Diagnosis of pylephlebitis is based on confirmatory imaging findings of portal vein or mesenteric vein thrombosis in the setting of systemic infection. Intravenous contrast-enhanced computerized tomography (CT), ultrasonography, or magnetic resonance imaging (MRI) can all be used to establish the diagnosis, with the former reported as the modality of choice given its availability and high sensitivity [28].

Pylephlebitis complications include thrombus extension into the superior mesenteric vein (SMV), thrombosis of the intrahepatic branches of the portal vein, liver abscesses, and inferior mesenteric vein thrombosis, in descending order of frequency [3]. Intestinal ischemia, a feared complication, can occur in 25% of patients with SMV thrombosis, carrying a mortality of 20% [3, 29]. Fortunately, the presence of a nonocclusive SMV thrombus in the reported case rendered the patient not at risk for intestinal ischemia; however, early recognition and treatment was critical to prevent progression. Long-term complications of untreated pylephlebitis include risks of chronic thrombus formation and resulting portal hypertension with associated complications of variceal bleeding and portal cholangiopathy [30].

Of the twenty-two cases of fusobacterium-related pylephlebitis, 41% involved solely the PV, 32% involved some combination of the PV, SMV, IMV, or splenic vein, and 14% involved the SMV alone. There was isolated involvement of the right hepatic vein in two cases, and in only one case was the IMV solely affected (Table 2). The variability in thrombosis extent, variable anticoagulation, and follow-up periods render assessing whether thrombus extent affects long-term outcomes limited.

The mainstay treatment of pylephlebitis is fluid resuscitation, antibiotics, and often anticoagulation [4]. Antibiotic choices can be guided by culture and sensitivity when available or empirically with broad-spectrum enteric and anaerobic coverage [20, 21]. Two large case series report similar mortality in patients treated with a combination of anticoagulation and antibiotics vs. antibiotics only [4, 5]. However, anticoagulation lowers the risks of future chronic portal hypertension and, hence, is frequently utilized in the absence of contraindications [5]. The length of anticoagulation utilized is a minimum of 3 months, but the optimal duration is unclear [5]. Choices of anticoagulation include unfractionated heparin in the acute setting later transitioned to low molecular weight heparin, warfarin, or direct oral anticoagulants (DOAC) [5, 31]. Treatment of *Fusobacterium-spp.*-associated pylephlebitis in the same vein follows the same principles. However, significant heterogeneity has been reported in use and duration of anticoagulation, where some authors anticoagulated patients only for the duration of their hospitalization while others anticoagulated patients between 3–9 months (Table 2). Given the absence of other risk factors for recurrent thrombosis in our patient, we elected to treat the event like a provoked deep vein thrombosis for a duration of 3 months.

Interestingly, while mortality has been reported as 11% in one case series of patients with pylephlebitis [4] and 12% in another [5], short-term outcome of patients with *Fusobacterium-spp.*-associated pylephlebitis appears more favorable with illness resolution in all cases where follow-up was reported (21 of 22 cases) and no cases of mortality including the reported case (Table 2). While this may represent a less fulminant course, it could be secondary to selection reporting biases that case reports are inherently prone to.

In conclusion, we present a case of pylephlebitis, a rare and often deadly complication, in the context of *Fusobacterium necrophorum* bacteremia. Our patient, whose presentation was mild and nonspecific, later developed septic shock. Adequate supportive therapy, prompt antibiotic therapy, and anticoagulation therapy with a DOAC for 3 months resulted in a favorable outcome. We hope that the presented case and review of existing cases contribute to the understanding of the subtle and insidious course of this rare complication.

## Figures and Tables

**Table 1:** Demographic and clinical characteristics and diagnostic imaging modalities in patients with *Fusobacterium-spp.*-associated pylephlebitis.

Author (year)	Age, \sex	Comorbidities	Symptoms	Bilirubin (mg/dl)	ALP (U/L)	AST (U/L)	ALT (U/L)	Source of infection	Imaging modality
Presented case	57, F	EtOH abuse and nonischemic CM	Fever, myalgias, and confusion	1.4	348	124	104	Colitis	MRCP
Abdallah et al. (2020)	37, M	Migraines	EtOH abuse fever, diarrhea, and abdominal pain	2.7	174	152	167	Diverticulitis	CT
Hamera et al. (2019)	51, M	UC, T2DM, and COPD	Fever and weakness	1.6	—	45	112	Cholangitis	US and CT
Le Roux et al. (2006)	43, M	EtOH abuse and pancreatitis	Fever and myalgias	NL	NL	NL	NL	No source identified	US and CT
Mellor et al. (2017)	64, M	None	Fever, diarrhea, and abdominal pain	3.4	234	—	—	Diverticulitis and pericolonic abscess	CT
Moore et al. (2016)	60, M	Not specified	Fever, epigastric pain, and weight loss	NL	NL	NL	NL	Unclear source	CT
Radovanovic et al. (2019)	69, M	HNSCC	Fever and abdominal pain	—	300	—	—	Liver abscess	CT and MRI
Rahmati et al. (2017)	59, F	Multiple sclerosis	Abdominal pain, fatigue, and weight loss	1.6	264	47	41	Hepatic abscess	CT and MRI
Tharu and et al (2020)	41, M	Diverticulosis	Fever and diarrhea	—	152	—	—	Hepatic and brain abscesses	CT
Akhrass et al. (2015)	32, M	None	Epigastric pain	NL	NL	NL	NL	Acute perforated appendicitis	US and CT
Zheng et al. (2014)	73, M	Hypertension, T2DM, and CAD	Fever and epigastric pain	NL	NL	NL	NL	No identified source	CT
Hamidi et al. (2008) case 1	23, M	None	Fever, epigastric pain, diarrhea, and jaundice	9.4	528	—	—	No identified source	YS
Hamidi et al. (2008) case 2	41, M	EtOH abuse	Fever, abdominal pain, and jaundice	5.3	—	254	59	No identified source	CT
Soo et al. (1999)	31, M	None	Fever, abdominal pain, diarrhea, and jaundice	5.9	295	79	133	No identified source	US and MRI
Shahani et al. (2011)	34, M	EtOH abuse and chronic pancreatitis	Epigastric pain	NL	NL	NL	NL	Hepatic, pancreatic, and splenic abscesses	CT
Clarke et al. (2003)	19, F	Previously healthy	Fever, abdominal pain, and jaundice	5.7	331	—	52	Hepatic abscesses	US and CT
Redford et al. (2005)	53, M	Previously healthy	Fever, abdominal pain, and vomiting	1.4	194	—	—	No identified source	CT
Bultink et al. (1999)	23, M	Previously healthy and oropharyngeal infection 5 weeks prior	Fever, abdominal pain, and vomiting	—	192	—	113	Possible pharyngitis	US and CT
Verna et al. (2004)	56, M	UC	Fever, anorexia, and jaundice	4.9	305	—	123	No identified source	CT
El Braks et al. (2004)	71, F	Urinary incontinence	Fever, sore throat, and epigastric pain	1.6	521	—	73	Pharyngitis	US and CT
Etienne et al. (2001)	68, M	TB and pulmonary embolism (not on anticoagulation)	Fever	—	—	—	—	Possible oropharyngeal source and concomitant liver abscess	CT
Schweigart et al. (2005)	67, M	TB, stroke, T2DM, and upper respiratory infection weeks prior	Fatigue, night sweats, and nausea	—	175	—	—	Possible oropharyngeal source	CT and MRI

Abbreviations: M: male, F: female, TB: tuberculosis, T2DM: type 2 diabetes mellitus, UC: ulcerative colitis, HNSCC: head and neck squamous cell cancer, COPD: chronic obstructive pulmonary disease, CAD: coronary artery disease, EtOH: alcohol, CM: cardiomyopathy, ALP: alkaline phosphatase, AST: asparatate aminotransferase, ALT: alanine aminotransferase, CT: computed tomography, US: ultrasound, MRI: magnetic resonance imaging, MRCP: magnetic resonance pancreaticography, NL: reported normal.

**Table 2:** Thrombus extent, management, and outcomes in patients with *Fusobacterium-spp.*-associated pylephlebitis.

Author (year)	Clot extent	Antibiotic regimen	Anticoagulation agent	Anticoagulation duration	Outcomes
Presented case	PV and SMV	I: vancomycin and meropenem F: ertapenem (2 weeks)	UFH and apixaban	3 months	Recovery and elective sigmoidectomy 2 years later
Abdallah et al. (2020)	PV, SMV, and IMV	I: meropenem F: ertapenem (1 month)	UFH and warfarin	6 months	Recovery and asymptomatic at 6-month follow-up
Hamera et al. (2019)	PV	I: meropenem F: ceftriaxone/metronidazole (1 month)	Enoxaparin	1 month	Recovery and no long-term follow-up
Le Roux et al. (2006)	SMV	F: amoxicillin-clavulanate and metronidazole (10 months)	UFH	Hospitalization	Recovery, 10-month follow-up, and abdominal US with chronic PVT
Mellor et al. (2017)	IMV	I: piperacillin-tazobactam F: ertapenem (1 month)	UFH	Hospitalization	Recovery, 1-month follow-up, and CT with PVT resolution
Moore et al. (2016)	Right PV	I: piperacillin-tazobactam F: metronidazole (4 weeks)	Enoxaparin and warfarin	6 months	Recovery and asymptomatic at follow-up
Radovanovic et al. (2019)	PV	I: ceftriaxone/metronidazole F: amoxicillin-clavulanate (2 weeks)	Enoxaparin and warfarin	3 months	Recovery and asymptomatic at 1-month follow-up
Rahmati et al. (2017)	PV	I: ertapenem F: ceftriaxone/metronidazole (2 months), ampicillin-clavulanate (2 months)	Enoxaparin	—	Recovery and 4-month follow-up CT with cavernous PV transformation
Tharu and et al. (2020)	Superior right hepatic vein	I: vancomycin/ceftriaxone/metronidazole F: ceftriaxone/metronidazole (6 weeks)	None	—	Recovery and 1-month CT with PVT resolution
Akhrass et al. (2015)	Main PV at the confluence	I: piperacillin-tazobactam F: Clindamycin (6 weeks)	UFH and warfarin	6 weeks	Recovery and follow-up not reported
Zheng et al. (2014)	Right hepatic vein	I: cefepime F: clindamycin	Enoxaparin and warfarin	—	Recovery and 2-month follow-up
Hamidi et al. (2008) case 1	SMV	I: amoxicillin-clavulanate (during admission) F: —	LMWH and fluindione	6 months	Recovery and 5-month follow-up US with PVT resolution
Hamidi et al. (2008) case 2	SMV	None	None	None	Left against medical advice
Soo et al. (1999)	PV andSMV	I: ciprofloxacin/metronidazole/penicillin F: amoxicillin-clavulanate/metronidazole (6 weeks)	UFH and warfarin	6 months	Recovery and 4-month MRI with resolved PVT
Shahani et al. (2011)	Left and right PV, SMV, and splenic vein	I: vancomycin/meropenem F: tigecycline (4 weeks)	None	None	Recovery and 2-month CT with cavernous PV transformation
Clarke et al. (2003)	PV and SMV	F: benzylpenicillin/metronidazole/ciprofloxacin (6 weeks)	UFH and warfarin	Indefinite	Recovery and 7-weeks US with PVT improvement
Redford et al. (2005)	PV	I: metronidazole/benzylpenicillin F: clindamycin (5 weeks)	LMWH and warfarin	3 months	Recovery and 3-months follow-up
Bultink et al. (1999)	PV	I: imipenem F: penicillin G (6 weeks)	IV heparin	Hospitalization	Recovery and 2-month US with chronic PVT
Verna et al. (2004)	Left PV	F: clindamycin (2 weeks)	None	None	Recovery and 6-month CT with chronic PVT and left hepatic atrophy
El Braks et al. (2004)	PV and SMV	F: piperacillin-tazobactam (2 weeks) and ofloxacin (3 weeks)	UFH and fluindione	9 months	Recovery and CT at follow-up (not reported) with chronic PVT
Etienne et al. (2001)	PV	F: cefotaxime and metronidazole (2 weeks) and metronidazole (2 weeks)	LMWH	3 weeks	Recovery and 5-week US with resolution
Schweigart et al. (2005)	PV	F: clindamycin	Warfarin	Indefinitely	Recovery and no long-term follow-up

Abbreviations: I: initial regimen, F: final regimen, PV: portal vein, SMV: superior mesenteric vein, IMV: inferior mesenteric vein, CT: computed tomography, US: ultrasound, MRI: magnetic resonance imaging, UFH: unfractionated heparin, LMWH: low-molecular-weight heparin, PVT: portal vein thrombosis.

## Data Availability

Data regarding this case/manuscript are available upon request from the corresponding author.
